# Current concepts in the management of high-energy tibial plateau fractures: a narrative review

**DOI:** 10.1530/EOR-2025-0167

**Published:** 2026-06-01

**Authors:** Nicolas Franulic, Marco Koch, José Tomás Muñoz, Rodrigo Olivieri, Nicolas Gaggero, Rodrigo Pesantez

**Affiliations:** ^1^ Knee Unit, Orthopedic Department, Hospital del Trabajador ACHS, Santiago, Chile; ^2^ Knee Unit, Orthopedic Department, Hospital Militar de Santiago, Santiago, Chile; ^3^ Faculty of Medicine, Department of Traumatology, Universidad de los Andes, Santiago, Chile; ^4^ Knee Unit, Orthopedic Department, Hospital El Carmen Dr Luis Valentin Ferrada, Santiago, Chile; ^5^ Orthopedic Department, Fundación Santa Fe de Bogotá, Bogotá, Colombia

**Keywords:** tibial plateau fractures, staged surgical treatment, three-dimensional fracture assessment, knee surgical approaches, prone surgical approach, posteromedial fragment management, posterolateral fragment management

## Abstract

- Tibial plateau fractures are complex intra-articular injuries where fracture morphology, trauma mechanism, and soft tissue condition critically influence treatment complexity and injury prognosis. Medial plateau fractures typically involve metaphyseal blocks with limited articular comminution, whereas lateral plateau fractures often present with greater fragmentation and depression.
- The Schatzker and AO/OTA classification systems are widely used, while those proposed by Luo, Krause, and Kfuri have enhanced the three-dimensional understanding and surgical planning of tibial plateau fractures.
- Associated soft tissue injuries can be divided into soft tissue coverage (skin, subcutaneous tissue, and muscle), which require early assessment and continuous monitoring, and structural injuries (ligaments and meniscus), which need more detailed evaluation and subsequent imaging studies.
- Open fractures and acute compartment syndrome are entities frequently associated with high-energy tibial plateau fractures. Early diagnosis and treatment – with early intravenous antibiotic prophylaxis, surgical debridement, prompt fasciotomies, and fracture stabilization – are needed to prevent further complications, such as fracture-related infections.
- Although immediate internal fixation can be safe in selected patients, a staged management approach with initial external fixation in complex or high-energy fractures enables soft tissue coverage healing while minimizing complications.
- The enhanced three-dimensional understanding of these fractures justifies a 360° surgical approach, addressing each fragment through specific approaches and fixation.

## Introduction

Tibial plateau fractures (TPFs) are complex intra-articular injuries with an even more arduous surgical management. The injury’s prognosis will be determined by fracture type, morphological characteristics, and the extent of soft tissue compromise, relating directly to the energy of the trauma itself (1, 2). Typically, high-energy fractures present with complex patterns, along with severe damage to the soft tissue envelope and/or neurovascular structures (3). Similarly, low-energy trauma may induce the same fracture characteristics in osteoporotic bone and may lead to considerable soft tissue damage (4, 5). Hence, initial clinical evaluation is paramount.

A staged surgical protocol has been proposed to treat urgent situations and prepare the patient, the fracture, and the surgical team for definitive surgery (6, 7). This allows a detailed imaging study of the fracture and associated injuries, given that soft tissue injuries may be present in around 93% of patients according to recent reviews (7, 8). It also provides time for the surgeon to fine-tune surgical planning and review special considerations, such as fracture morphology, plausible knee position at the time of the injury, and feasible plate positioning. Hyperextension injuries are deemed of particular attention as they are highly associated with neurovascular or ligament injuries with poor results (9). Despite meticulous planning, complications after surgical treatment are relatively high with high-energy trauma injuries, resulting in poor long-term functional outcomes (3, 6). Patients must be warned of fracture-related infection (FRI), malunion, and post-traumatic osteoarthritis – among others – as possible future complications (3, 10).

The objective of this article is to present a narrative review of the current literature on the approach and management of high-energy TPFs, information which could also be utilized in the management of low-energy fractures with complex patterns or a great extent of soft tissue injuries.

### Classifications

The initial classifications of TPFs were primarily based on the interpretation of standard anteroposterior radiographs (10). In 1974, Schatzker and colleagues proposed one of the most widely recognized classification systems, comprised of six principal fracture patterns arranged in order of increasing severity (11). In 2018, Kfuri and Schatzker revisited this original classification and underscored the relevance of accurately determining the three-dimensional location of the displaced fragment or fragments to guide both the surgical approach and the specific placement of fixation plates (12).

With the advent of computed tomography (CT), Luo *et al.* (13) introduced a classification that incorporates the concept of anatomical columns. Subsequently, in 2016, Krause *et al.* (14) proposed a segment-based classification in which the fracture pattern was analyzed through a systematic mapping of the tibial plateau. To optimize surgical planning, the tibial plateau was divided into anterior and posterior columns on the axial plane, and into central, medial, and lateral sections on the coronal plane. These classification systems enable a more precise assessment of the involved anatomical zones and facilitate the selection of the most appropriate surgical approaches.

Additionally, isolated medial TPFs have been further subclassified by Wahlquist *et al.* (15). Their system divides medial plateau fractures into three categories – A, B, and C – according to whether the fracture line exits medial, within, or lateral to the intercondylar eminence, respectively (15). This classification is both easily remembered and widely recognizable.

There is no single ideal classification. However, Schatzker’s classification has been widely recognized, along with others such as the Luo *et al. *(13), Krause *et al. *(14), and Kfuri & Schatzker classifications (12), which allow for surgical planning optimization. Knowledge of the aforementioned classification systems allows surgeons to have a better understanding of the diverse TPFs and their proper management.

### Energy and injury mechanism

The energy of the accident is one of the most important factors to determine fracture patterns, as well as knee angulation and bone density. These elements will collectively produce a specific fracture pattern (16).

High-energy trauma leads to more comminution and unpredictable fracture patterns (5). A high complication rate has been reported in the literature for this specific group of patients (17) due to not only the Schatzker high-energy patterns – IV, V, and VI– but also the immense degree of soft tissue damage (12).

Knee position – from flexion to extension – and injury force vector – from varus to valgus – will determine the extent of compromise of a specific articular segment (16). A recent article published by Luo *et al.* describes the main fracture characteristics according to the mechanism of injury (Table 1) (16). A detailed understanding of mechanism-associated fracture patterns may enhance the planning and execution of fracture reduction and fixation, such as performing a maneuver that opposes the inferred mechanism of injury. In this regard, in the setting of posteromedial split-type fractures resulting from a flexion–varus mechanism, a hyperextension and valgus technique may facilitate reduction. Conversely, following this same rationale, in instances of posterolateral depression fractures (flexion–valgus group), knee extension combined with varus can improve fracture-site exposure and assist in achieving reduction (16).

**Table 1:** Fracture morphological characteristics by injury mechanism, as listed by Xie *et al.* (16).

Injury mechanism	Fracture morphological characteristics
Flexion and varus	Posteromedial fragment with posterior wedge apex
Flexion and valgus	Posterolateral fragment with or without posterior rim disruption
Extension and varus	Large medial fragment with apex either medial or symmetrically divided anterior and posterior in the tibial metaphysis
Extension and valgus	Lateral central depression and/or split fragment
Hyperextension and varus	Anteromedial depression with adjacent rim disruption
Hyperextension and valgus	Anterolateral depression with adjacent rim disruption

It is essential to identify the specific articular segments involved in each fracture. According to the study by Krause *et al.* (14), which describes the frequency of involvement of the ten defined articular segments, the distribution of compromised areas varies consistently between OTA/AO type B and type C fracture patterns. In type B fractures, the lateral tibial plateau was the most frequently involved segment (88.8%), either alone (85.1%) or in combination with the medial plateau (3.7%). The most common comminution patterns involved the combined anterior and posterior segments (65.2%). Moreover, the three most frequent segment combinations accounted for 41% of all described variants, including pure depression, split-depression, and complete plateau fractures. Among these, the most prevalent combination involved nearly the entire lateral tibial plateau, sparing only the tibial spine, most often affecting the anterior, lateral, or posterior cortex (14). In type C fractures, a greater diversity of fracture patterns and segment combinations was observed. In this group, involvement of the tibial spines and central segments was common (89.4%). Comminution zones were more frequently located in the lateral plateau, whereas the medial plateau more often demonstrated split-type fractures with distinct anterior and posterior fragments (14).

This is consistent with what has been previously reported in the literature. Reduced bone density may predispose complex fracture morphology even in low-energy mechanisms (18). In line with this, it is important to highlight that, due to load distribution, the lateral tibial plateau has lower bone density, which – together with its more convex morphology – predisposes it to articular comminution, depression, and widening (14). In contrast, the medial plateau bears approximately 60% of the load, resulting in a thicker and more resistant subchondral bone (14). Consequently, and in combination with its more concave morphology, the medial plateau is more prone to split-type fractures at the metaphyseal level, characterized by shear components and minimal articular comminution. In this regard, although medial plateau fractures may occur in low-energy mechanisms (14), they are more commonly associated with higher-energy trauma, making thorough assessment of soft tissue compromise essential (5).

Another increasingly recognized fracture pattern and mechanism is the hyperextension-type injury. These fractures are characterized by the presence of anteromedial/anterolateral compression patterns, tension failure of the posterior cortex of the proximal tibia, and inversion of the tibial slope in the sagittal plane (19). They may also present as compressive and marginal fractures of the anterior rim, lacking cortical containment. Although these account for less than 20% of bicondylar fractures, their relevance lies in their association with poorer functional outcomes (19, 20). This is largely attributable to the difficulty of achieving proper reduction, the high rate of associated injuries, and the limited bone stock typically present in these fractures, along with insufficient cortical support at the anterior rim, which makes the use of traditional implants challenging (19). Additionally, these fractures are accompanied by a high rate of associated injuries (over 32%), including vascular injuries to the popliteal artery, neurologic compromise of the common peroneal nerve and acute compartment syndrome (ACS) (20), and ligamentous injuries involving the posterolateral corner and the posterior cruciate ligament (21). A classification that may help in understanding their morphology and guiding treatment is the one proposed by Yao *et al.* (22), which is based on the involvement of the anterior segments, assessed in the coronal plane, and on the extent of the fracture line toward the posterior cortex.

### Soft tissue evaluation

Soft tissues around the knee are of paramount importance and may evolve quickly over time when related to high-energy accidents. Management of TPFs requires good preoperative planning for soft tissues and the fracture itself, considering it ‘a soft tissue injury with a fracture related to it’ (23). Favorable clinical results will be obtained only if soft tissue care and management are successful.

Soft tissue evaluation and management may be divided into two groups: injuries of tissue surrounding the knee or coverage injuries (considering skin, subcutaneous, and muscle) and ligament/meniscal injuries. Coverage injury may be apparent in initial clinical examination, but ligament and meniscal injuries require critical evaluation afterward.

#### Coverage injuries

Initial trauma triggers an inflammatory response that leads to edema, venous stasis, dermal hypoxia, and ischemic damage to the soft tissue envelope (23). This process may favor the development of blisters, dermal, and even muscular necrosis, directly compromising the soft tissues surrounding the fracture (23).

A thorough evaluation of both osseous and soft tissue injuries, along with well-structured sequential surgical planning, is essential for successful treatment. Initial management should focus on preventing soft tissue deterioration through fracture immobilization and cryotherapy (24). Definitive fracture reduction and fixation may be postponed until the soft tissue condition is optimal. External fixation can be used as a temporary method for fracture reduction and stabilization through ligamentotaxis, thereby promoting soft tissue recovery (24, 25). The surgical technique and other relevant aspects of external fixation in TPFs are described in the ‘External fixation’ section. In many cases, the appropriate timing for definitive fixation involves waiting 7–14 days, allowing sufficient time for the soft tissues to recover and regain normal turgor (24). This recovery can be clinically assessed using the ‘wrinkle sign.’

In some cases, despite external fixation and other soft tissue care measures, fracture blisters may still develop. Several management strategies have been described. Many surgeons prefer to maintain the integrity of the blisters while waiting for the soft tissues to recover, typically covering them with a non-adherent dressing beneath standard dressings. If the blisters have ruptured, they may be completely unroofed, usually in the operating room, and the raw blister bed covered with a non-adherent dressing (24).

#### Ligament and meniscal injuries

Several studies have reported a high prevalence of ligament and meniscal injuries associated with high-energy TPFs in magnetic resonance imaging (MRI) (26, 27, 28). The stated prevalence may vary because of the heterogeneity of the fractures included in the different studies.

A systematic review published by Frosch and Krause, including 18 studies and 877 patients, reported a 93% prevalence for all ligament and meniscal injuries (8). A prevalence of 48.9 for lateral meniscal injury, 36.8% for anterior cruciate ligament rupture, 24.5% for medial meniscal injury, 22.9% for lateral collateral ligament injury, 20.7% for medial collateral ligament injury, and 14.8% for posterior cruciate ligament rupture was reported. As stated by the authors, and to our knowledge, to date, no study has established an exact association between different fracture types and morphology, and soft tissue injuries of ligament or meniscus, making the generation of unique treatment algorithms somewhat challenging (8). The proper identification of these injuries is crucial for achieving good postoperative results (29).

### Imaging study

The traditional imaging study was based on simple anteroposterior, lateral, and oblique radiographs. It is common knowledge that this type of study may not be sufficient, as it does not allow for correct fragment identification. The use of CT is recommended for this type of fracture. Chan *et al.* observed that the use of routine CT not only enhances the inter- and intraobserver correlation at the time of classifying these injuries but also generates a change in surgical planning in up to 26% of occasions (30). Similar findings were reported by Macarini *et al.* and Fleming *et al.* (31, 32). They observed that CT modified the initially proposed treatment in 60 and 28.5%, respectively. CT allows a better fracture morphology interpretation, fragment identification, and articular depression characterization (33). With the popularization of CT, many authors have stated the real importance of this imaging technique in articular fractures (33).

**Figure 1:**
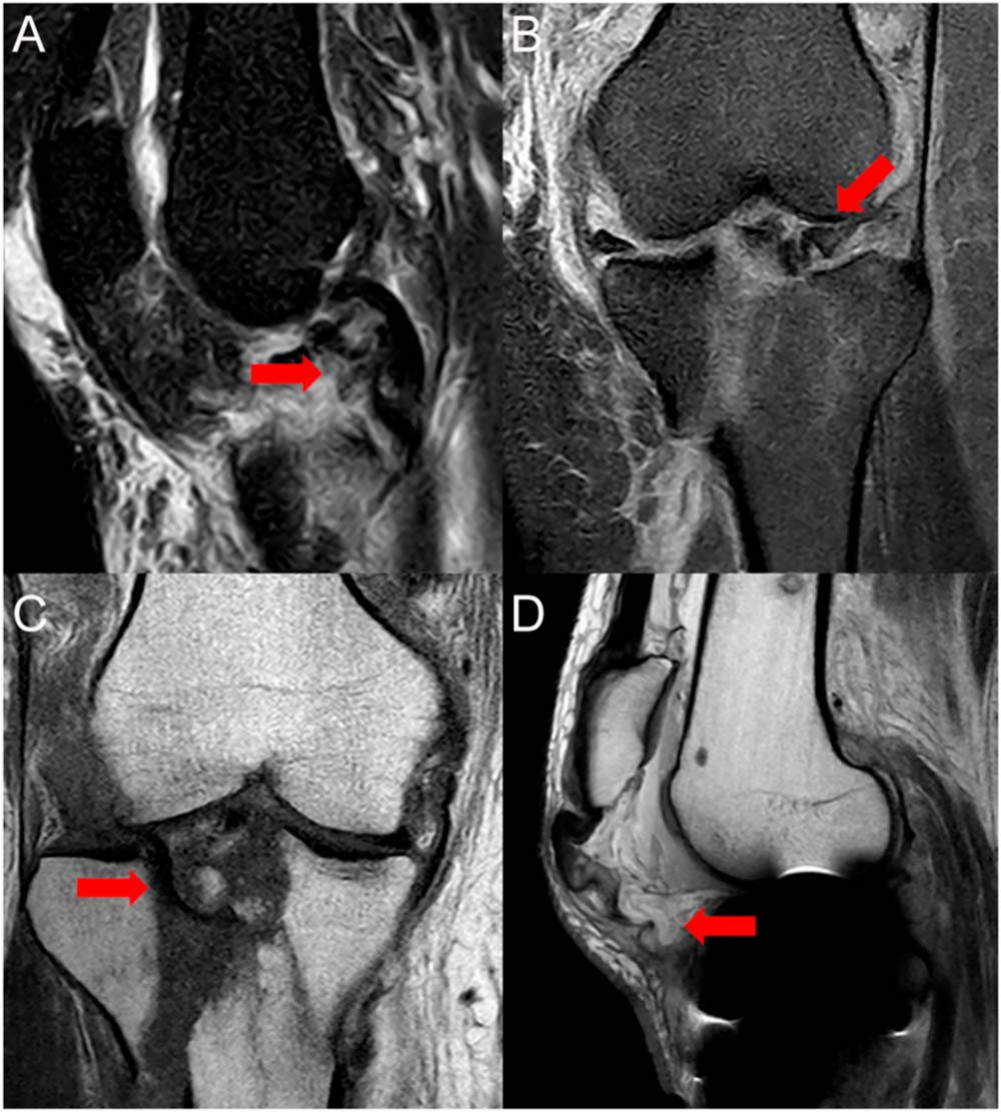
MRI may be useful to identify intra- and extra-articular pathology that may require concomitant surgery. (A) Bucket-handle tear of the lateral meniscus displaced into the intercondylar notch (sagittal plane). (B) Bucket-handle tear of the lateral meniscus displaced into the intercondylar notch (coronal plane). (C) Lateral meniscus interposed within the fracture site, preventing proper reduction of the fracture. (D) Confirmation of patellar tendon detachment from the anterior tibial tuberosity.

MRI is useful to assess associated soft tissue damage (34, 35). Many authors propose the systematic use of MRI in the evaluation of high-energy TPFs (36). A systematic review published by Krause *et al.* in 2023 (8) suggests that MRI may be useful in high-energy trauma, knee fracture-dislocation patterns, posterolateral fractures, and tibial plateau widening (8). Crawford *et al.* analyzed the diagnostic accuracy of MRI, comparing it to knee fracturoscopy, reporting that MRI may be preferable to arthroscopy in the majority of cases, being a safer and prompt study that identifies intra- and extra-articular pathology that may require concomitant surgery – as Stener-type injuries, meniscal injuries, or ruptures of the extensor mechanism (37) (Fig. 1).

If MRI is not available, the imaging study with radiographs or CT may allow the surgeon to infer soft tissue injuries and address them in the surgical intervention. Many studies have found an association between some fracture characteristics and soft tissue injuries (38, 39). Gardner *et al.* described a higher prevalence of lateral meniscal injuries if patients had an articular depression of more than 6 mm or a lateral tibial plateau widening of more than 5 mm on simple radiographs in comparison with those who did not (83 vs 50%, respectively) (26). Studies relating some values and signs related to meniscus injuries in CTs have also been performed. Tang *et al.* described a significant association between lateral plateau depression >11 mm and the presence of a lateral meniscus lesion. Also, they reported that a medial plateau displacement >3 mm is associated with an elevated risk of suffering an avulsion fracture of the anterior cruciate ligament (40). Likewise, Salari *et al.* reported that with every 1 mm of impaction/displacement in the articular surface, there is a 21% increased risk of sustaining a meniscus injury and that with an articular impaction/displacement of 4.3 mm has a 100% sensibility in the detection of lateral meniscal tears (41). Finally, proximal fibular fractures (PFFs) are another important factor to address in imaging studies. PFFs have been associated with a higher probability of meniscus injury and as an indicator of high-energy trauma (42).

### Open fractures

Open fractures have an incidence of 4–43% of TPFs and are classified through the Gustilo–Anderson classification (25). Open fractures have been reported as one of the most relevant factors for the development of infection related to TPFs (43, 44, 45, 46). A recent multicentric study published in 2023 reported a 5.5 times higher risk of the development of FRI in their multivariate analysis (47). Although there is also published literature in which open fractures did not reach statistical significance for FRI (48, 49), their management via early intravenous antibiotic prophylaxis, surgical debridement, and fracture stabilization seems like a feasible approach in order to prevent FRI (50, 51).

There is still debate regarding the ideal moment for the definitive treatment of open high-energy TPFs. In 2005, Egol *et al.* described their results with staged management using an external fixator, reporting a low rate of complications and proposing staged management – associated with an exhaustive study and planification of the fracture – as the appropriate strategy, while waiting for the soft tissues to heal in order to proceed with definitive fixation (25). Nevertheless, authors like Virkus *et al.* have stated that the risk of complications after staged management is not significantly different compared to acute open reduction and internal fixation (50). In patients with open TPFs, a novel study published in 2023 reported no significant differences between definitive early fixation (<24 h after the trauma) and delayed staged treatment regarding the development of FRI and unplanned reoperations (51). The same authors described significantly higher rates of FRI (83.3 versus 16.7%) and reoperation (71.4 versus 33.3%) in patients with open TPFs of Gustilo–Anderson types III B and C, in which the final flap coverage exceeded 7 days after the definitive fixation, compared to early flap coverage (51). Considering early coverage of soft tissue defects is paramount in the management of open TPFs.

### Compartment syndrome

The association of ACS and high-energy TPFs is well known. It develops in 4.3–14.5% of TPFs (52). Delayed diagnosis of ACS is associated with inferior clinical outcomes (45, 52, 53) and, if not treated, may lead to disastrous consequences – from loss of function to life-threatening conditions or death (52, 53). Clinical suspicion upon presentation is fundamental in order to prevent ACS, in particular in polytrauma patients or those with impaired consciousness state that may not present the classic cardinal symptoms and signs as unreasonable pain or pain to passive range of motion of joints distal to the fracture.

The presence of risk factors may alert the clinician to the possible debut of ACS. Risk factors can be divided into radiographic and non-radiographic (53). Younger age (12–29 years old), male gender, high-energy trauma, and ballistic injuries account for the non-radiographic risk factors (53). Open fractures have not been identified as a protective or predictive factor for ACS. Regarding radiographic fractures, Schatzker types IV and VI, and OTA/AO 41-C. Concomitant tibial shaft, fibular fractures, longitudinal fracture extension, and higher tibial widening/femoral displacement have been listed as potential risk factors (53). Yet – to consider – the largest available published series failed to find an association between tibial widening/femoral displacement and ACS (54). In their article, Marchand *et al.* highlighted high-energy mechanisms, such as Schatzker type VI fractures, concomitant tibial shaft or fibular fractures, and fracture length, as potential risk factors for the development of ACS (54). They reported that patients with 3 and 4 predictive factors presented 20 and 27% risk of developing ACS, respectively (54).

**Figure 2:**
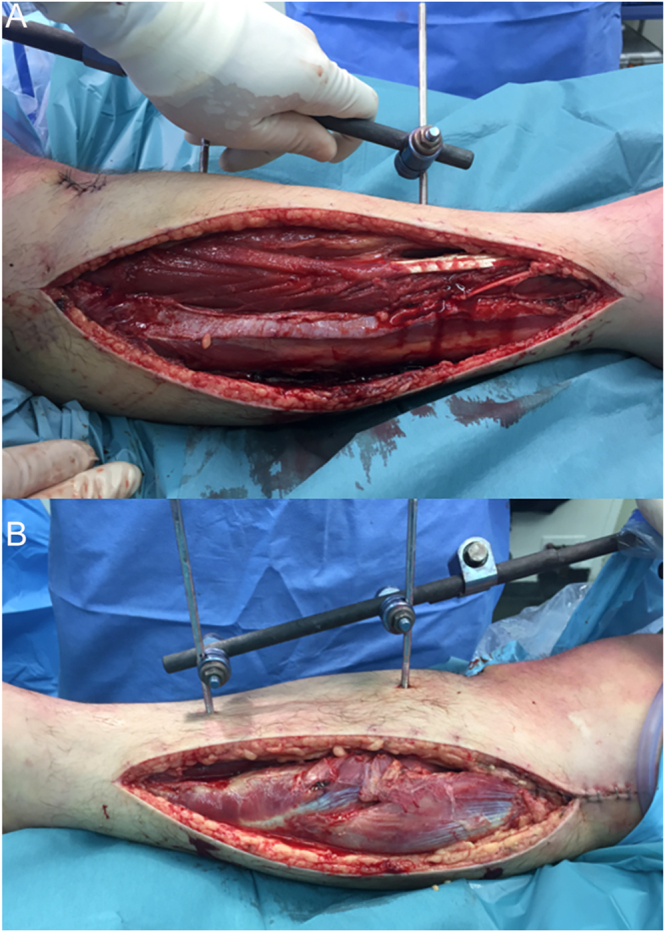
Lateral and medial leg fasciotomies for the management of ACS associated with a high-energy TPF.

ACS is a medical emergency and – as such – its treatment must be initiated at the time of suspicion. Management of combined high-energy TPFs with ACS must be centered on functional reduction and external stabilization, along with 4-compartment leg fasciotomies. One or two incisions can be performed, as described by many authors (55, 56) (Fig. 2). No differences regarding FRI have been reported between single or dual incision fasciotomies (55). After liberation, fasciotomies must be managed carefully through sequential surgical debridement and techniques that allow the closure of the wounds. Elastic closure via vascular ties or negative pressure wound therapy (NPWT) has helped the closure of fasciotomy wounds (55, 56, 57). Sequential surgical debridement must be performed every three to five days, in order to evaluate the muscular tissue integrity and excise nonviable tissue. Wound closure must be achieved with no tension and in the absence of muscular necrosis. At least three days before closure are recommended after initial surgery, as anticipated closure has been related to increased intramuscular pressure (53).

Concern about ACS in patients with high-energy TPFs arises due to the alleged increased risk of FRI (46, 58). However, the association remains controversial, and there is evidence that contradicts it (59, 60). To date, the only systematic review and meta-analysis published by the group of Shao *et al.,* including 2,214 patients with a prevalence of 9.9% for FRI, reported an OR of 3.53 for the surgical site infection in TPFs with ACS (58).

Another aspect to consider is the timing of fasciotomy wound closure. Although Zura *et al.* (60) reported no significant differences with respect to the moment of fasciotomy wound closure and FRI, there is a great amount of literature that supports the closure of wounds before definitive fixation, as closure at the moment or after the definitive osteosynthesis may increase the risk of FRI (52, 61, 62, 63). A multicentric retrospective cohort study published by Dubina *et al.* described a 16% FRI in simultaneous fasciotomy wound closure, in comparison to 20.5% if closed before and 21.8% if closed after the definitive surgery (52). An increased risk of infection of 7% for every day of delayed closure has been reported by the same group (62). Likewise, Ruffolo *et al.* reported an OR 7.5 for patients with closure during the definitive fixation or after it (61). Similar results are described by Henkelmann *et al.* in a recent multicentric study (63). Although the literature appears to support wound closure before or at the time of internal osteosynthesis, there is a special concern of not delaying the definitive surgical intervention, as this may decrease the surgeon’s capacity to accomplish anatomic articular reduction. This was reported in a novel study published in 2024 by Flagstad *et al.* (64).

### Neurovascular injuries

Common peroneal nerve (CPN) injury is the most common neurologic injury of the lower extremities, with a relatively high incidence of 16–40% in knee dislocations (65). There is a paucity of evidence on the type of injuries related to high-energy TPFs. To our knowledge, the case series reported by García-Fernández *et al.* is the largest available, reporting a 1.47% CPN injury in closed TPFs (66). Ninety-one percent of the fractures that presented CPN injuries showed a varus deformity on initial radiographs, supporting the theory that varus displacement injures the nerve via traction and entrapment at the fibular neck (66). Concomitant fibular head fracture was present in 56% of patients, and this may be a sign that may alert the clinician in patients with medial or both column fractures (66). Franulic and colleagues recently reported an incidence of neurological injuries of 4.06% (67). Of these patients, 84.6% sustained high-energy trauma. Similarly, proximal fibular fractures were present in 61.5% of neurological cases, showing an association between the presence of a proximal fibular fracture and neurologic injury (OR 4.46).

In relation to vascular injuries, a prevalence of approximately 1.04% has been reported after lower-extremity fractures (68). Specifically regarding TPFs, Franulic *et al.* described in their series of 320 patients an incidence of 0.62% (2 of 320 patients), which is the largest series reported, highlighting that all patients were evaluated with CT angiography (67). Both cases of vascular injury occurred in patients with high-energy trauma, open fractures, and fracture patterns classified as Schatzker VI and AO/OTA C3. To the above-mentioned literature, only case reports can be added, describing ruptures, transections, and occlusions of the popliteal artery in patients with TPFs (69, 70).

**Figure 3:**
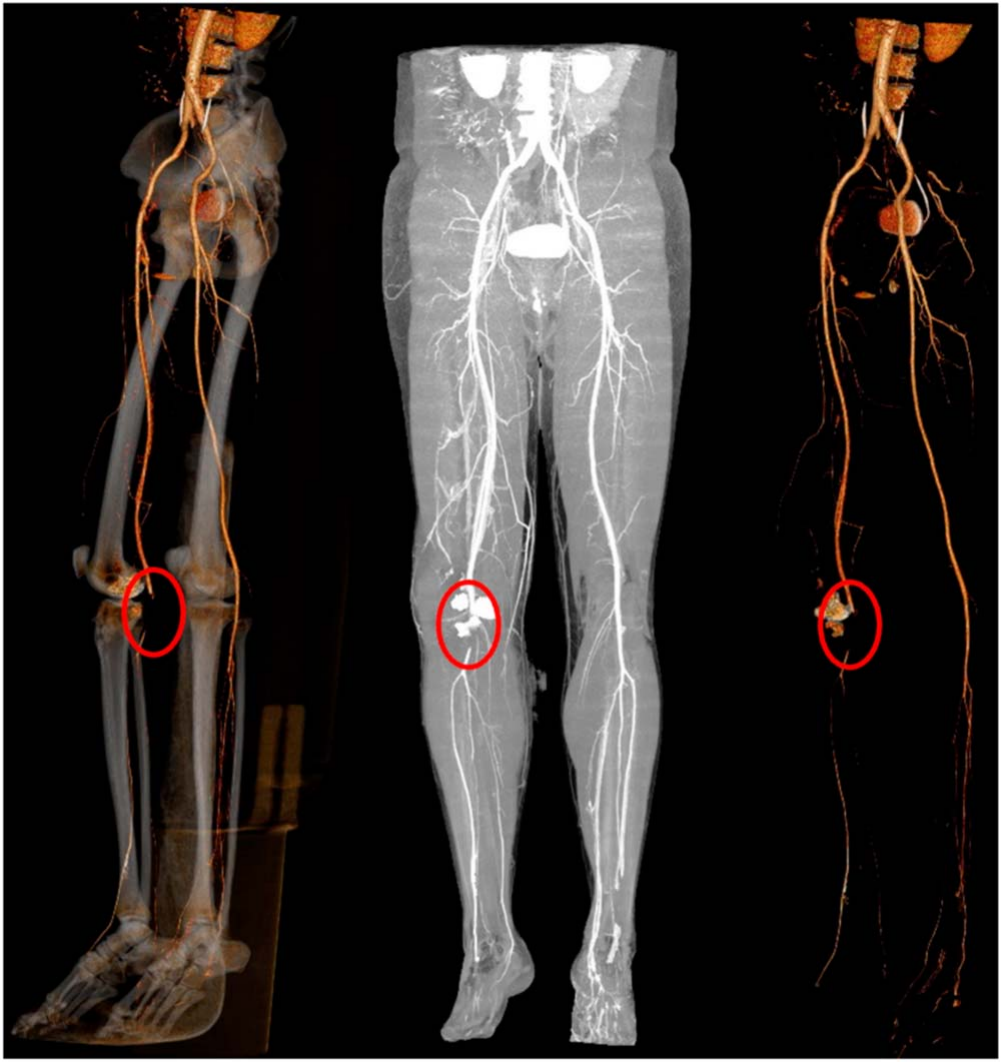
A high-energy TPF with a 4 cm popliteal artery occlusion.

Even though physical examination has been highlighted as one of the most useful tools in the diagnosis of vascular injuries, in some cases, collateral vascular circulation may alter the results and produce no clinical findings even if the popliteal artery is damaged (71). If hard signs of vascular injury are present upon initial evaluation, revascularization must be performed immediately. An ankle-brachial index (ABI) < 0.9 is highly indicative of vascular insult, although it is technically demanding as it requires Doppler ultrasound and sequential controls when performed adequately. The use of CT angiography of lower extremities has become relevant and allows the surgeon to identify vascular injuries in a rapid and cost-effective alternative (71) (Fig. 3).

### External fixation

Initial treatment of high-energy TPFs should focus on preventing greater damage to soft tissue. Immobilization is crucial in order to decrease the inflammatory response (24). The surgeon can achieve this by using an external spanning fixation, most commonly indicated for patients with open fractures, ACS, neurovascular injuries, or high-energy TPFs (4). For proper positioning of the spanning external fixator, two anterolateral pins in the femur and two anteromedial pins at the tibia – to avoid the fracture site and future surgical approaches – are recommended (24). It is important to consider a multiplanar configuration to add more stability and use carbon rods that allow correct fracture characterization. After installation and proper fracture reduction, axial traction is applied before tightening.

However, the introduction of internal fixation implants motivated some surgeons to perform immediate open reduction and internal fixation (ORIF) (72, 73, 74). Yet, surgical complications, such as FRI, malunion, non-union, and arthrofibrosis, seen as high as in 88% of cases turn the balance toward delayed definitive fixation and initial external spanning fixation.

A classic study published by Egol *et al.* (25) evaluated external fixation for transient fixation in 53 patients with high-energy TPFs (OTA type 41) and described a low surgical site infection (<5%) in patients with staged management but with an increased probability of subsequent arthrofibrosis. The authors recommend staged management of high-energy TPFs, open fractures, and ACS (25). Similarly, Giordano *et al.* described good results with staged treatment, allowing the first hit of inflammatory response remission and adequate surgical planning (1). A more recent study published in 2020 by Barwar *et al.* concluded that staged treatment favors soft tissue healing, preventing complications such as FRI and ACS without altering fracture reduction, union rates, or functional outcomes (75). Other studies have reported similar outcomes, with low rates of soft tissue complications, FRI, skin necrosis, and satisfactory functional and radiographic outcomes (76). The increased risk of FRI related to external spanning fixation has been refuted by many authors (51, 58, 77).

There is available literature that advocates immediate ORIF in bicondylar TPFs (72, 73, 74). One study of two level I trauma centers retrospectively evaluated 186 patients and reported inferior operative time in the immediate ORIF groups (157 vs 213 min, *P* < 0.001) and no significant differences regarding wound complication, FRI, non-union, reoperation, or post-traumatic arthritis (74). Surgical site infections and open fractures were higher in the group of staged treatment. The authors declared that immediate ORIF must be performed only in a selected group of patients, as it may not be appropriate for all high-energy TPFs (74). Kim *et al.* reported similar results in their retrospective study of 31 patients, with no significant differences in wound complication or reoperations, but described longer hospital stay and increased cost in the staged management group (73).

Even though there is a great amount of literature that describes no association between external spanning fixation and FR (43), there are some authors who reported opposite results and described external fixation as a risk factor for FRI (48, 73). These results must be taken with particular caution because, generally, patients with staged treatment are related to other risk factors, such as soft tissue injuries, high-energy trauma, and prolonged surgical time.

Some authors have described that pin-plate superposition has no relation with an increased risk of FRI (78, 79). Others, such as Shah *et al.*, reported a greater risk of infection in their series of tibial plateau and pilon fractures (80). Another study retrospectively analyzed 244 patients and reported surgical site infection in 27.7% in the superposition group and a relative risk of 3.01 (81). The same authors described that installing the tibial pins >100 and 150 mm from the fracture site decreased pin-plate overlap in 51 and 96% cases, respectively (82). Also, Stenquist *et al.* (83) and Moon *et al.* (81) reported no significant differences if the pins are removed at the same time as definitive fixation or previously.

Considering the above-mentioned evidence, immediate definitive treatment or staged treatment is a valid option, and the controversy is yet to be elucidated. To consider, external fixation can be the definitive fixation, as reported by some authors (77, 84, 85).

### Surgical approaches and specific fragment management: the 360° concept

Some years ago, TPFs were approached via anterior incisions and mainly by anterolateral or anteromedial plates. Nowadays – due to the better understanding of fracture morphology, specific fragments, and their relevance – the 360° concept has taken special importance in the management of TPFs. This concept includes performing varied incisions oriented by specific fragments (anterior or posterior) and the utilization of diverse plate constructs with different objectives and functions (4, 86, 87, 88, 89).

This particular change in vision and understanding has been provided by the new classifications described above, integrating posteromedial and posterolateral fragments, the anterior tibial tubercle, and tibial spines. In such a manner, Molenaars *et al.* described the tomographic characteristics of 127 TPFs, depicting four recurrent patterns: lateral split fragment (75%), posteromedial fragment (43%), comminuted tibial spine fragment with lateral extension (28%), and anterior tibial tubercle fragment (16%) (90). The authors emphasize the importance of considering these fragments in surgical planning in order to obtain satisfactory outcomes.

Building upon this understanding, when planning the surgical approach, it is essential to conceptualize TPFs in quadrants, as described by Schatzker–Kfuri (12, 91), Luo *et al*. (13), and Krause *et al*. (92). Each approach in our arsenal, as will be described below, serves as a distinct tool to address the specific compromised quadrant and type of fragment. The orientation of the main fracture plane dictates the placement of the hardware and the choice of our surgical approach (12).

Lateral split fragment is generally associated with articular depression, and anatomical reduction is directly related to good functional outcomes (93). The anterolateral approach with submeniscal arthrotomy is the surgeon’s workhorse when managing this specific fragment, allowing direct visualization of the articular fragments and their reduction via an anterolateral buttress plate. It should be highlighted that plate screws must be positioned near the articular surface in order to prevent fragment subsidence (94, 95). Cannulated screws may be considered with a minimally invasive incision if there is no articular depression or in particular occasions with previous percutaneous reduction (96).

The posteromedial fragment is generally big and has no articular comminution, and so, direct articular visualization is not necessary (94). The final position of the posteromedial plate and the selected approach will depend mainly on the exit of the fracture at the metaphyseal–diaphyseal zone level (4, 10). Prone and supine positioning approaches have been described in order to achieve a correct posteromedial fragment reduction and fixation (13, 97, 98, 99, 100, 101). In the particular situation of articular comminution, some authors have described a submeniscal arthrotomy to aid visualization and articular reduction (102, 103).

There is a paucity in the literature respecting the adequate management of the anterior tibial tubercle fragment. Maroto *et al.* recommend using cortical screws if there is no damage to the posterior cortex and a single large fragment. Comminuted fractures or posterior cortex incompetence are better treated using a plate and screws construct (104).

The management of posterolateral fractures has taken on special importance in recent years. Posterolateral fractures can be particularly difficult to approach due to the interposition of the fibular head that prevents correct visualization and reduction of the fragment. Initially, fibular head or lateral epicondyle approaches were described in order to resolve these issues (101, 105, 106, 107, 108, 109, 110, 111, 112). Nowadays, we have multiple surgical approaches that allow the surgeon to perform anatomical fracture reduction through small windows without requiring osteotomies (113, 114, 115, 116, 117, 118, 119, 120, 121, 122) (Table 2). The groups of Cho *et al.*, Chen *et al.,* and Frosch *et al.* – later modified by Mancini *et al.* – are particularly useful (114, 115, 116, 117, 118). Rim plates have revolutionized this fracture management and aid the surgeon in achieving correct fracture reduction and do not interfere with other osteosyntheses (91, 112, 114, 115, 123, 124, 125, 126). Rim plates have also been proposed in the management of posteromedial or anteromedial comminuted fractures (103, 127).

**Table 2:** Surgical approaches for posterolateral fragment management.

Study	Patient positioning	Osteotomy	Approach	Benefits	Limitations
Lobenhoffer *et al.* (101)	Supine/lateral decubitus	Fibular osteotomy	Longitudinal incision from LE to a point halfway between ATT and fibular head	Great exposure of the PL plateau. Concomitant fixation of AL fractures	Considerable soft tissue trauma; risk of NV injury; osteotomy nonunion; loss of reduction
Carlson *et al.* (100)	Prone decubitus	No	S-shaped posterior incision	Direct exposure of PL and posteromedial fractures. Reduction of the posterior plateau condyles is easiest with the knee in full extension.	Challenging; flexion contracture
Bowers *et al.* (106)/Kfuri *et al.* (112)	Supine/lateral decubitus	Lateral epicondyle osteotomy	Curvilinear incision from proximal to the LE to GT	Extensile approach; direct visualization; concomitant fixation of AL fractures	Osteotomy nonunion; challenging
Chen *et al.* (116)	Supine decubitus	No	S-shaped incision from the leading edge of biceps femoris directed to GT and ends 1 cm lateral to ATT	Avoids osteotomy and risk of NV injury; concomitant fixation of AL fractures	Difficult reduction of comminuted fractures; not suitable for fractures near the fibular head
Cho *et al.* (114)	Supine decubitus	No	Curvilinear incision along GT	Adequate exposure of AL and PL plateau without significant risk of NV injury; concomitant fixation of AL fractures	Difficult to buttress
Frosch *et al.* (117), modified by Mancini *et al.* (118)	Lateral decubitus	No	Longitudinal PL skin incision following the direction of the fibula; modified: S-shaped with the fibular head in the center (following the course of the CPN)	Direct visualization and manipulation through windows	Not extensible; challenging; need for CPN dissection and manipulation

LE, lateral epicondyle; ATT, anterior tibial tubercle; PL, posterolateral; AL, anterolateral; NV, neurovascular; GT, Gerdy’s tubercle; CPN, common peroneal nerve.

Conversely, hyperextension fractures constitute a particular entity, for which the key radiographic features and diagnostic criteria have already been described above. Their treatment goals include correcting the inverted tibial slope, reestablishing cortical support at the anterior rim, and achieving sufficient construct stability – particularly in cases where the fracture line extends completely to the posterior cortex, creating a true hinge (19, 20, 21). Although no standardized management algorithm exists, owing to their low frequency and relatively recent recognition, several principles proposed by Firoozabadi and Pires can help guide treatment (19, 20). Among them, in cases with complete slope inversion and posterior cortical failure under tension, the use of parallel K-wires directed toward the articular surface can assist in restoring the slope, combined with fixation at the posterior fulcrum and an anterolateral plate. The use of blade plates has also been described, and rim plates remain a valuable option when cortical support at the anterior margin is deficient (19, 20).

Finally, arthroscopy is a useful tool and may aid the surgeon in the diagnosis of associated injuries and articular reduction. It was initially described and limited to the management of low-energy TPFs because of the historic fear of ACS development after the procedure (128, 129). To consider, a recent study retrospectively analyzed 288 high-energy TPFs and reported no significant differences in complications between patients in whom arthroscopic assistance was needed and those who did not (130).

### Authors’ approach

**Figure 4:**
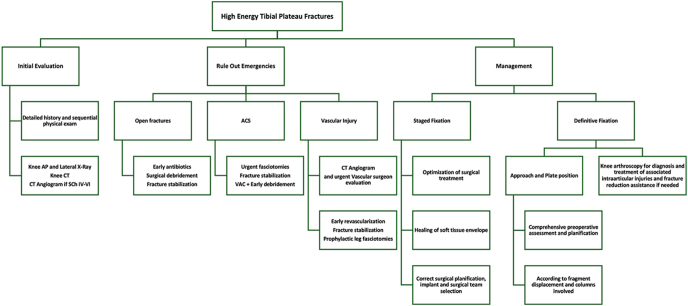
Algorithm proposed for high-energy TPF management.

As a group, we emphasize that management of high-energy TPFs needs particular attention to detail and it is different from the aid that low- or moderate-energy TPFs require. It is essential to have a structured and organized understanding of the factors to consider and the management to be implemented (Fig. 4). Knowing and understanding the injury mechanism is essential as it allows the surgeon to adopt an initial attitude toward the fracture and the management of polytrauma patients and the special care of evolving soft tissue conditions. We believe that sequential physical examination is paramount.

In every patient with suspicion of a high-energy TPF, our initial study includes anteroposterior and lateral knee radiographs and a knee CT. In our center, in every TPF classified Schatzker IV or higher, a CT angiogram is indicated as part of our institutional protocol. To date, there is no evidence that endorses the cost-effectiveness of this protocol, so it has to be evaluated according to your own care center conditions.

**Figure 5:**
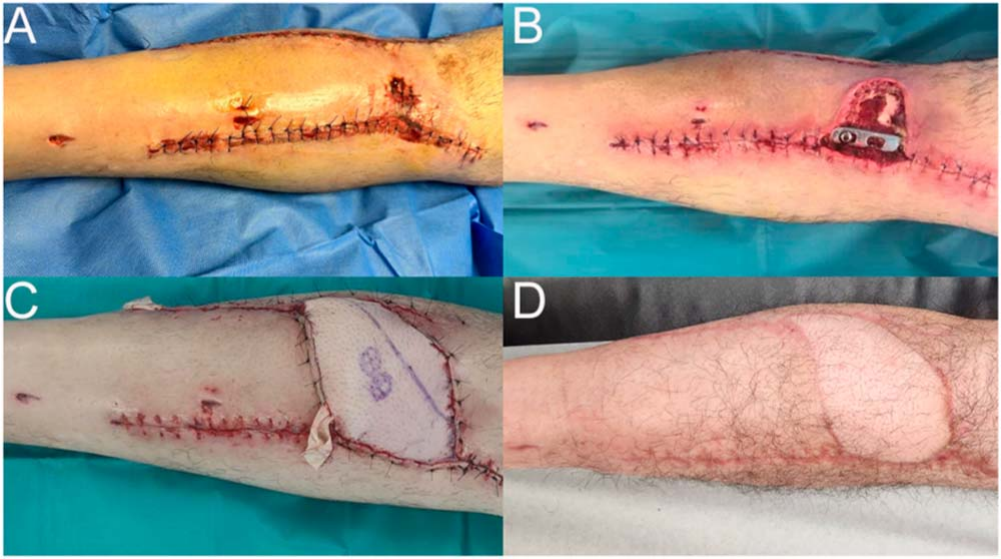
Patient with an open fracture of the tibial plateaus and concomitant compartment syndrome in the leg. (A) Wounds following surgical debridement of the exposed fracture and subsequent closure of leg fasciotomy incisions. (B) Wound dehiscence and loss of skin coverage after reduction and internal fixation of the TPF. (C) Coverage achieved with a free flap performed by the plastic surgery team. (D) Successful integration and healing of the free flap.

Open fractures are always treated with immediate antibiotic prophylaxis (cefazolin 2 g every 8 h and gentamicin 3–5 mg/kg once a day, for 72 h). Prompt surgical debridement is mandatory, and we recommend performing it as soon as possible, ideally <24 h after the trauma. Thorough surgical debridement and excision of nonviable tissue are essential in order to achieve the absence of macroscopic contamination and a clean fracture bed. In the situation of Gustilo–Anderson type III B fractures with soft tissue coverage defects and after surgical debridement, photographs are taken before installing a NPWT. Our center has a plastic surgery team specialized in the closure of these injuries, ideally before 7 days have elapsed from the initial trauma, as recommended by evidence (51) (Fig. 5).

In the situation of ACS, immediate surgical intervention is indicated. First, we address the fracture with external spanning fixation, which may aid the surgeon in better understanding the lower extremity anatomy, as it may be altered by the initial deformity and inflammatory response. Immediately, after achieving functional reduction, we proceed to a dual incision fasciotomy. We suggest considering future surgical approaches that the fracture may need before performing the fasciotomies, as they may be continued with the same incisions. We always use dual incision fasciotomies and never perform minimally invasive fasciotomies in a high-energy trauma context (Fig. 2). In the presence of altered anatomy, correct exposure and visualization are fundamental. NPWT is installed in all fasciotomy wounds. Surgical debridement is performed every 3–5 days in order to analyze tissue viability and excise all nonviable tissue. Wound closure is performed as soon as possible if the skin can be sutured with no tension. We do not suture the fascia. We recommend closing the medial wound first due to the absence of soft tissue coverage. After wound closure, we perform the definitive surgical fixation only if the local and systemic conditions of the patient allow the surgery.

As mentioned above, all high-energy TPFs are studied with CT angiography because we acknowledge that early revascularization of vascular injuries may decrease the risk of complications and salvage the extremity. However, we do not recommend this for all institutions, as there is no literature available on the cost-effectiveness of this stance. If surgical revascularization is needed, an external fixator is installed, and then a vascular surgeon performs the revascularization at the same time as our surgeon aids with dual fasciotomy incisions in order to prevent reperfusion ACS. A rapid and correct external spanning fixation is essential to prevent damage to the revascularization.

Regarding fracture reduction and fixation, there is literature that supports either immediate fixation (72, 73, 74) or staged fixation (1, 25, 76). Generally, we prefer the staged fixation protocol. Initial external fixation allows our surgeons the adequate treatment of polytrauma patients, a complete study of fracture characteristics and associated injuries (including MRI whenever possible), permitting correct planification, implant, and surgical team selection. Correct pin positioning is essential, which is appropriately illustrated in the article published by Borelli in 2013, which may be used as a guide for proper external fixation installation (24). Also, we emphasize the importance of preventing pin tract infection and standardizing local protocols, as evidence on this subject is scarce to make a proper recommendation (131, 132). In our center, femoral pins are cleaned daily and tibial pins every 2 days. If any subtle signs of pin tract infection are identified, the frequency of the cleaning is increased and the patient is assisted by topical mupirocin ointment. Oral or intravenous antibiotics and pin replacement are defined by a multidisciplinary approach by the knee surgeon and the infection department.

**Figure 6:**
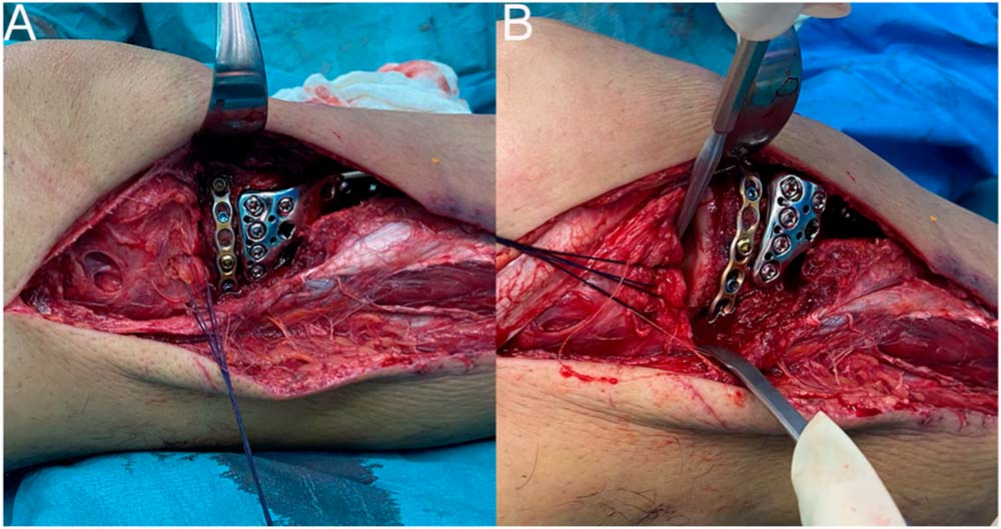
Posterolateral fragment management using the para-fibular collateral ligament space described by Cho *et al.* (114).

For definitive internal fixation, we consider that the surgeon’s knowledge of supine and prone positioning approaches is crucial, as it aids prompt reduction and fixation of specific fragments. We believe that posteromedial fragments are better managed by prone positioning approaches, and, if deemed necessary, we begin the surgery in this manner. For minimally displaced posteromedial fractures, a posteromedial approach with supine positioning with knee flexion and hip external rotation is preferred. For the management of posterolateral fragments, our group has a tendency to use the surgical approach described by Cho *et al.* (114) (Fig. 6). We believe that this approach is relatively simple (as it is way similar to the classical anterolateral approach) and has the advantage of not needing additional osteosynthesis (as it does not require additional osteotomies). Additionally, this approach has a low risk of iatrogenic CPN injury.

If needed, we use arthroscopy for the management of associated injuries such as anterior cruciate ligament avulsion injuries and meniscal repair (that cannot be performed through our approach) or for optimization of articular reduction. To date, we report no complications directly related to the use of arthroscopy in TPFs.

## ICMJE Statement of Interest

The authors declare that they do not have any conflict of interest that could be perceived as prejudicing the impartiality of the work reported.

## Funding Statement

This research did not receive any specific grant from any funding agency in the public, commercial, or not-for-profit sector.
